# Supplementary material for: Population-level HIV incidence estimates using a combination of synthetic cohort and recency biomarker approaches in KwaZulu-Natal, South Africa
Source: PLoS One. 2018 Sep 13;13(9):e0203638. doi: 10.1371/journal.pone.0203638 (PMC6136757; doi:10.1371/journal.pone.0203638)
Supplement: S1 Appendix — (PDF) [file pone.0203638.s001.pdf]

S1 Appendix: Optimal RITA identification and calibration

The data used in the calibration and contextual adaptation of the Recent Infection Testing Algorithm (RITA) used in this study were provided by the CEPHIA Group. The UCSF Human Research Protection Program and IRB (formerly CHR, #10-02365) approved the study procedures.

The performance of a test for recent infection or RITA for the purposes of incidence estimation is captured in two parameters: The Mean Duration of Recent Infection (MDRI) and the False-Recent Rate (FRR).

The *MDRI* is the average amount of time that individuals spend exhibiting the 'recent' biomarker, while infected for less than some cut-off time (denoted T, 2 years in the present work). This captures the defining biological aspects of the recency test. The *FRR* is the proportion of individuals infected for longer than the cut-off time T, but who nevertheless produce a recent result on the test. FRR is inevitably context-dependent, and critically depends on epidemiological factors such as the prevalence of HIV infection and antiretroviral treatment coverage. The latter is important, because with the reduction in antigenic pressure associated with viral suppression, immune markers tend to revert to a state similar to early infection, resulting in 'false' recent classifications. Inclusion of a viral load threshold in a RITA reduces the FRR in treated individuals to close to zero.

In order to estimate MDRI and context-specific FRR for a given RITA, the chosen recent infection case definition was applied to LAg, Bio-Rad Avidity and viral load results on the CEPHIA evaluation panel. The CEPHIA evaluation panel consists of 2,500 well-characterised specimens and was employed in the independent evaluation of a range of candidate tests for recent infection, including both the LAg and Bio-Rad Avidity assays. In order to define an 'optimal' RITA, a range of combinations of thresholds on three available biomarkers – the LAg normalised optical density (ODn), the Bio-Rad Avidity index (AI) and the viral load (copies/ml) – were applied and MDRI and context-dependent FRR estimated for the epidemiological context of this study.

MDRI was estimated by fitting a regression model for the probability of testing recent as a function of estimated time since infection, $P_R(t)$, using a logit link function and a cubic polynomial in time (since estimated date of detectable infection), to CEPHIA evaluation panel data. The function was fit to data points up to 800 days post-infection. The MDRI was then obtained by integrating the function from 0 to T. Confidence intervals were obtained by resampling subjects in 10,000 bootstrap iterations. MDRI was estimated on subtype C-infected specimens only, the predominant subtype in the surveyed population. The estimated MDRI was adjusted for the sensitivity of the screening algorithm used in this study, namely NAAT in pools of five specimens (effective detection threshold of 500 copies/ml), i.e. 7.7 days shorter MDRI than estimated using the CEPHIA reference test [1].

Estimating *context-dependent FRR* requires defining the epidemiological context, namely HIV prevalence, HIV incidence and treatment coverage and then estimating FRR in untreated and treated individuals separately, combining the estimates into a weighted average according to treatment coverage in the population. To obtain contextual epidemiological parameters, the survey data was analysed to obtain prevalence and treatment coverage proportions, and an initial incidence analysis was

conducted, using a standard RITA comprising LAg (≤ 1.5) and viral load (> 100 copies/ml), with MDRI and a crude FRR estimated from CEPHIA data, to obtain an overall incidence in the population of interest. ARV testing was not included in the RITA, as this may have an unknown impact on MDRI, and earlier work using this survey demonstrated limited benefit for FRR and precision of incidence estimates [2].

These parameters were then employed to estimate context-specific FRR, by estimating the FRR in untreated individuals and treated individuals separately, and weighting these estimates according to treatment coverage. Confidence intervals were obtained by resampling subjects in 20,000 bootstrap iterations. For untreated individuals, the function $P_R(t)$ was fit using CEPHIA data for subtype C-infected individuals, from all times post-infection, and weighted according to the probability density function for times since infection in the untreated population. The distribution of times since infection was parameterised as a Weibull survival function (i.e. remaining in the untreated state), with the shape and scale parameters chosen to produce the desired treatment coverage in a population with the specified incidence and prevalence and scaled to recent incidence. The FRR in treated subjects, $P_{R|tx}$ is simply the binomially estimated probability that treated subjects infected for longer than T would produce a recent result,[1] since the FRR in treated subjects appears not to depend strongly on time since infection. The weighted FRR estimate was obtained as shown in Eq 1:

$$\epsilon_T = c \cdot P_{R|tx} + (1 - c) \cdot \int_T^{\inf} \rho(t) P_R(t)\, dt \tag{1}$$

where c is the treatment coverage, $\rho(t) = \frac{f(t)}{\int_T^{\inf} f(t)\, dt}$, $f(t) = \exp(-(\frac{t}{\alpha})^\beta)$ and α and β the Weibull scale and shape parameters, respectively. This approach was previously described in [3].

We obtained incidence estimates for a range of LAg and Bio-Rad Avidity threshold combinations, together with a viral load threshold of 75 copies/ml, and evaluated relative standard error (RSE) on the incidence estimate. Reproducibility of the incidence estimate was obtained from 100,000 bootstrap iterations, each drawing from the distributions of test property estimates, HIV prevalence and prevalence of recency among HIV-positives (from the survey dataset, analysed using the survey R package [4] to account for the complex sampling frame), using the inctools R package [5]. We adopted the ‘optimal’ recency case definition of: (NAAT-positive and antibody negative) OR (antibody positive, LAg ODn ≤ 2.5, Bio-Rad Avidity AI ≤ 30 and viral load > 75 copies/ml). A selection of threshold combinations is shown in Table 1.

References

1. Grebe E, Facente S, Powrie A, Gerber J, Priede G, Chibawara T, et al.. Infection Dating Tool; 2018. Online application. Available from: https://tools.incidence-estimation.org/idt/.

2. Huerga H, Shiferie F, Grebe E, Giuliani R, Farhat JB, Van-Cutsem G, et al. A comparison of self-report and antiretroviral detection to inform estimates of antiretroviral therapy coverage, viral load suppression and HIV incidence in

[1] In the CEPHIA Evaluation Panel, all treated subjects were virally suppressed, resulting in an estimate of $P_{R|tx} = 0$ in all cases where a supplemental viral load threshold is applied. In real-world populations, it is likely that a certain (unknown) proportion of treated subjects would be virally unsuppressed and that the FRR in treated subjects would therefore be non-zero.

Table 1. Precision of incidence estimate produced by a range of LAg, Bio-Rad Avidity and viral load threshold combinations.

**LAg ODn** $\leq$	**Bio-Rad AI** $\leq$	**Viral Load** $>$	**MDRI*** (95% CI) *days*	**Context-adapted FRR** (95% CI) %	**RSE on incidence** %
1.5	20	75	156 (136,179)	0.06% (0.00%,0.17%)	18.90%
1.5	30	75	172 (148,197)	0.06% (0.00%,0.17%)	18.70%
1.5	40	75	176 (152,201)	0.12% (0.01%,0.31%)	19.20%
1.5	50	75	179 (155,205)	0.12% (0.01%,0.31%)	19.20%
1.5	60	75	179 (155,205)	0.13% (0.01%,0.33%)	19.40%
1.75	20	75	161 (140,183)	0.09% (0.02%,0.22%)	18.20%
1.75	30	75	178 (155,204)	0.09% (0.02%,0.22%)	18.00%
1.75	40	75	186 (160,215)	0.16% (0.02%,0.39%)	18.70%
1.75	50	75	190 (164,219)	0.16% (0.02%,0.40%)	18.70%
1.75	60	75	193 (166,223)	0.17% (0.03%,0.41%)	19.10%
2	20	75	172 (149,196)	0.09% (0.02%,0.22%)	18.00%
2	30	75	191 (166,218)	0.09% (0.02%,0.22%)	17.80%
2	40	75	200 (174,229)	0.16% (0.02%,0.39%)	18.40%
2	50	75	205 (177,235)	0.16% (0.02%,0.39%)	18.50%
2	60	75	208 (179,238)	0.18% (0.02%,0.41%)	18.70%
2.25	20	75	186 (164,210)	0.11% (0.03%,0.25%)	17.20%
2.25	30	75	210 (184,236)	0.12% (0.03%,0.27%)	17.10%
2.25	40	75	222 (192,254)	0.21% (0.05%,0.47%)	18.20%
2.25	50	75	232 (201,265)	0.22% (0.05%,0.47%)	18.10%
2.25	60	75	237 (206,269)	0.42% (0.08%,1.14%)	20.50%
2.5	20	75	194 (171,219)	0.14% (0.04%,0.31%)	16.80%
**2.5**	**30**	**75**	**217 (192,244)**	**0.17% (0.05%,0.35%)**	**16.60%**
2.5	40	75	233 (204,265)	0.30% (0.09%,0.61%)	17.90%
2.5	50	75	249 (218,282)	0.30% (0.10%,0.61%)	17.60%
2.5	60	75	260 (227, 293)	0.51% (0.15%,1.26%)	19.30%

*MDRI adjusted for sensitivity of screening algorithm

Kwazulu-Natal, South Africa. BMC Infectious Diseases. 2017;17(1):653. doi:10.1186/s12879-017-2740-y.

3. Grebe E, Welte A, Hall J, Keating SM, Facente SN, Marson K, et al. Infection Staging and Incidence Surveillance Applications of High Dynamic Range Diagnostic Immuno-Assay Platforms. Journal of Acquired Immune Deficiency Syndromes. 2017;76(5):547–555. doi:10.1097/QAI.0000000000001537.

4. Lumley T. survey: analysis of complex survey samples; 2017. R package version 3.32. Available from: `https://cran.r-project.org/package=survey`.

5. Welte A, Grebe E, McIntosh A, Bäumler P. inctools: Incidence Estimation Tools; 2018. R package version 1.0.11. Available from: `https://cran.r-project.org/package=inctools`.
