# Supplementary material for: Population-level HIV incidence estimates using a combination of synthetic cohort and recency biomarker approaches in KwaZulu-Natal, South Africa
Source: PLoS One. 2018 Sep 13;13(9):e0203638. doi: 10.1371/journal.pone.0203638 (PMC6136757; doi:10.1371/journal.pone.0203638)
Supplement: S2 Appendix — (PDF) [file pone.0203638.s002.pdf]

S2 Appendix: Sensitivity analyses

Sensitivity of incidence estimates to False-Recent Rate

To evaluate the sensitivity of incidence estimates to the false-recent rate (FRR), we repeated the estimation procedure using values of FRR ranging from 0% to 1%. With higher FRR values, the biomarker-based estimates of incidence are lower, and this impact is greatest at the higher ages (which also have higher prevalence). Fig 1 shows the age-continuous biomarker-based estimates for demonstrative ages (ages 15, 20, 25 and 30) for both males and females. For this analysis a relative standard error (RSE) on the FRR estimate of 50% was assumed (similar to the estimated value used in the primary analysis).

Estimates using the combined method for FRR values ranging from 0% to 1% and RSE on FRR of 50% are shown in Fig 2. In these plots the incidence estimates counter-intuitively do not decline with higher FRRs. This results from greater uncertainty in the biomarker-based estimates, owing to the large RSE on FRR, which results in the combined estimates being weighted towards the synthetic cohort-based estimates (which are higher). To avoid this artefact, the analysis was repeated assuming an RSE on FRR of 0%, shown in Fig 3.

Fig 1. Incidence estimates (biomarker method) for a range of FRR values (relative standard error on FRR of 50%).

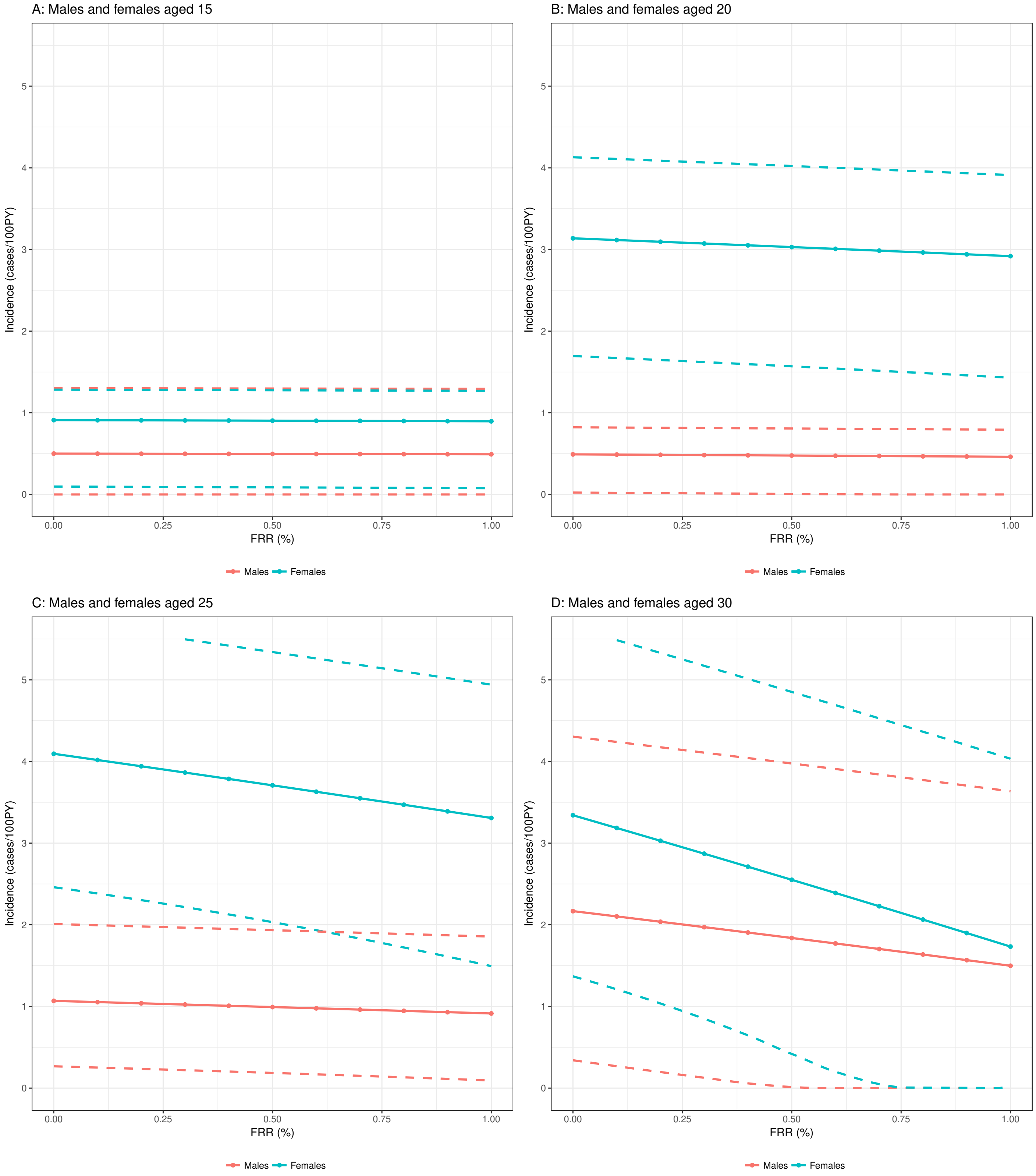

Fig 2. Incidence estimates (combined method) for a range of FRR values (relative standard error on FRR of 50%).

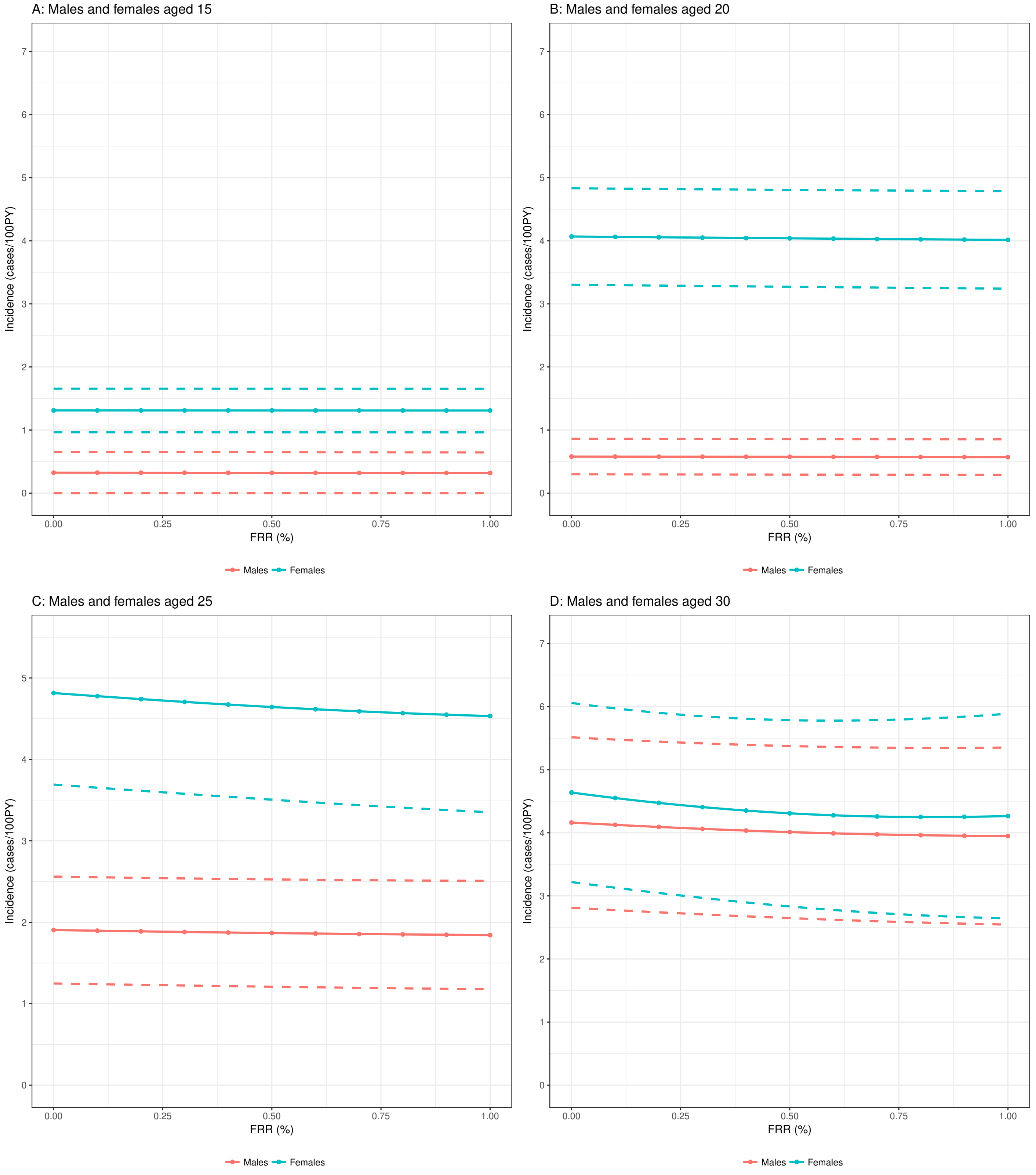

Fig 3. Incidence estimates (combined method) for a range of FRR values (relative standard error on FRR of 0%).

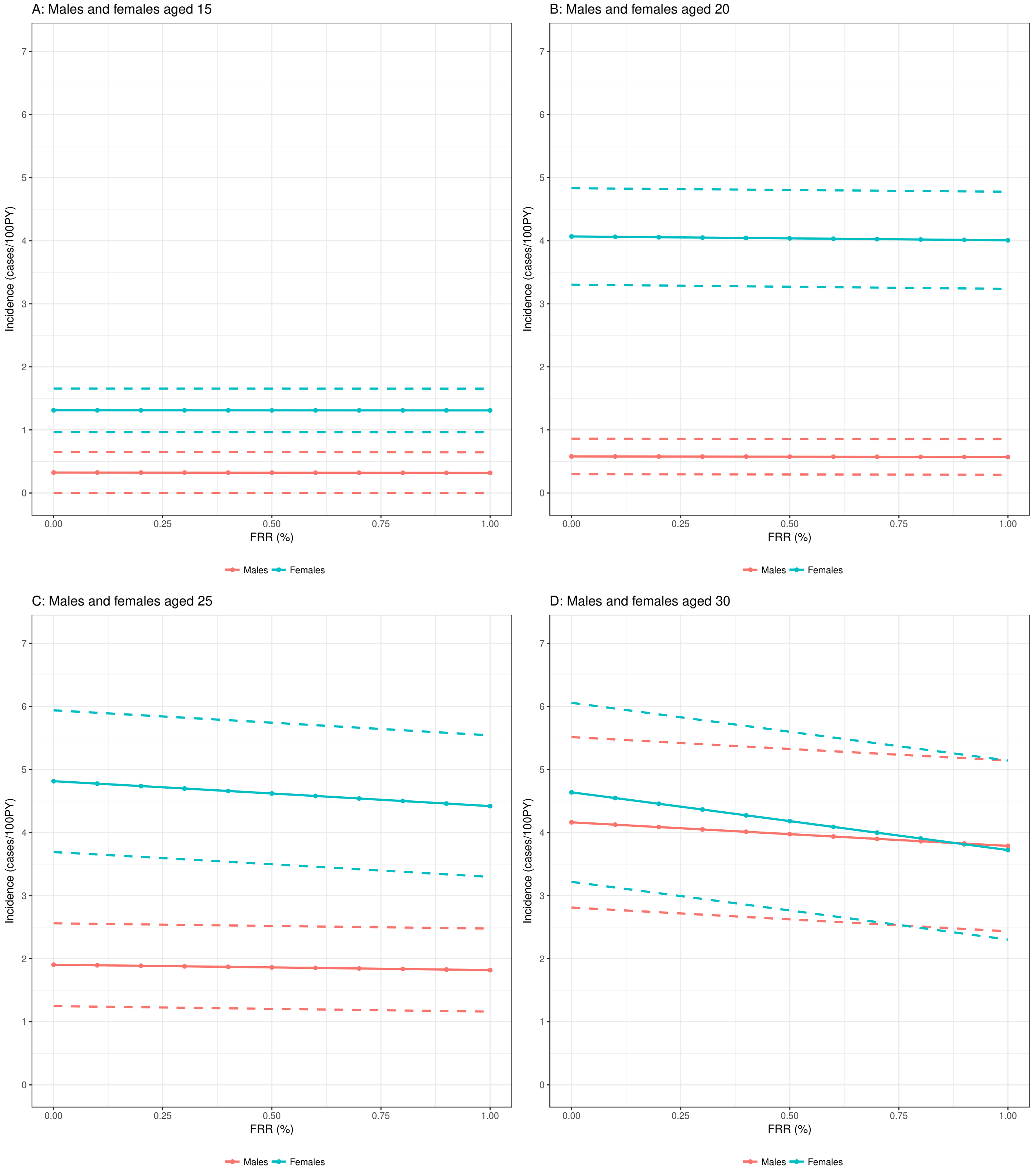

Sensitivity of average incidence estimates to weighting scheme

Calculating 'average incidence' over a particular age range requires estimating sex and age-specific incidence and weighting the estimates over the age range by either the sampling density or the susceptible population density (see Methods section). In Table 1, we report the average incidence obtained for a set of age ranges when weighting by the sampling or susceptible population densities. Only the susceptible population-weighted estimates are reported in the main text of the paper.

Table 1. Average incidence estimates (combined method), weighted by susceptible population density and sampling density.

	**Weighted by susceptible population density**			**Weighted by sampling density**		
**Age group**<br>*years*	**Males**<br>P.E. (95% CI)<br>*cases/100PY*	**Females**<br>P.E. (95% CI)<br>*cases/100PY*	**Total**<br>P.E. (95% CI)<br>*cases/100PY*	**Males**<br>P.E. (95% CI)<br>*cases/100PY*	**Females**<br>P.E. (95% CI)<br>*cases/100PY*	**Total**<br>P.E. (95% CI)<br>*cases/100PY*
[15,20)	0.36 (0.00,0.61)	2.62 (2.07,3.11)	1.51 (1.13,1.75)	0.37 (0.04,0.61)	2.77 (2.17,3.28)	1.46 (1.09,1.69)
[20,25)	1.22 (0.71,1.49)	4.73 (4.00,5.54)	2.95 (2.46,3.28)	1.16 (0.67,1.42)	4.72 (3.98,5.51)	2.94 (2.46,3.27)
[25,30)	2.64 (1.13,3.26)	4.65 (3.68,5.79)	3.61 (2.48,4.19)	2.72 (1.16,3.36)	4.65 (3.67,5.81)	3.79 (2.65,4.37)
[30,35)	2.76 (0.38,4.7+9)	3.56 (1.77,6.15)	3.14 (1.38,4.65)	2.88 (0.48,4.64)	3.62 (1.89,6.11)	3.29 (1.54,4.73)
[15,30)	1.26 (0.64,1.49)	3.83 (3.35,4.37)	2.54 (2.07,2.77)	1.14 (0.55,1.36)	3.94 (3.41,4.51)	2.52 (2.09,2.77)

As can be seen in Table 1, average incidence estimates for 5-year age bins, as well as the entire 15-30 age group are not very sensitive to weighting scheme. In all cases the point estimate for average incidence weighted by sampling density falls within the 95% confidence interval of the susceptible population density-weighted estimates, and the differences in point estimates are very small.

Sensitivity of incidence estimates to time-varying age structure of prevalence

We have no direct information from the cross-sectional survey on the prevalence gradient in time (i.e. the partial derivative of prevalence with respect to time), as required by the original Mahiance et al. synthetic cohort incidence estimator:

$$\lambda(a,t) = \frac{1}{1-p(a,t)} \cdot \left(\frac{\partial}{\partial t}p(a,t) + \frac{\partial}{\partial a}p(a,t)\right) + \delta(a,t) \cdot p(a,t) \tag{1}$$

We therefore repeated the entire estimation procedure, assuming various age-specific prevalence gradients in time, $\frac{\partial}{\partial t}p(a,t)$, at the time of the survey. The THEMBISA model's age-specific prevalence estimates for the period 2010 to 2014 indicate a stable epidemic in persons aged 15-35, with approximately linear change and gradients varying between -0.009 and 0.010 in females and between -0.013 and 0.004 in males in the various one-year age bins.

We parameterised assumed change in age-specific prevalence by age as an exponential decline (or increase) in the form $p(a,t) = p(a) \cdot e^{-\gamma t}$ so that $\frac{\partial}{\partial t}p(a,t) = -\gamma \cdot p(a,t)$. A steep decline in prevalence (halving of prevalence in approximately 10 years) is obtained with $\gamma = 0.07$. We repeated the analysis for males, females and overall (using data for all ages, but reporting results for age-specific incidence at ages 15, 20, 25 and 30) using four values, representing rapidly increasing and decreasing age-specific prevalence and more plausible time-gradients of prevalence, as well as the extreme case where prevalence is plummeting (with a half-life of approximately 5 years, $\gamma = 0.14$):

$$\gamma \in \{-0.07, -0.035, 0.035, 0.07, 0.14\}$$

The results, compared to the primary analysis, assuming $\frac{\partial}{\partial t}p(a,t) = 0$, are shown in Table 2.

Table 2. Age-specific incidence estimates (combined method) at a range of values for γ.

Age	Sex	Incidence P.E. (95% CI) $\frac{\partial}{\partial t}p(a,t) = 0$	Incidence P.E. (95% CI) $\gamma = -0.07$	Incidence P.E. (95% CI) $\gamma = -0.035$	Incidence P.E. (95% CI) $\gamma = 0.035$	Incidence P.E. (95% CI) $\gamma = 0.07$	Incidence P.E. (95% CI) $\gamma = 0.14$
15	Males	0.32 (0.00,0.65)	0.34 (0.03,0.65)	0.33 (0.01,0.65)	0.32 (0.00,0.65)	0.31 (0.00,0.65)	0.30 (0.00,0.65)
20	Males	0.58 (0.30,0.86)	0.68 (0.37,0.99)	0.63 (0.34,0.93)	0.52 (0.25,0.78)	0.45 (0.19,0.70)	0.30 (0.06,0.55)
25	Males	1.89 (1.23,2.55)	2.03 (1.35,2.72)	1.97 (1.30,2.64)	1.78 (1.14,2.42)	1.64 (1.01,2.28)	1.31 (0.68,1.93)
30	Males	4.10 (2.75,5.46)	4.58 (3.10,6.05)	4.41 (2.99,5.83)	3.62 (2.33,4.91)	2.98 (1.73,4.22)	1.44 (0.20,2.68)
15	Females	1.31 (0.97,1.66)	1.42 (1.01,1.83)	1.37 (0.99,1.75)	1.24 (0.93,1.56)	1.17 (0.88,1.46)	1.01 (0.75,1.26)
20	Females	4.06 (3.29,4.82)	4.79 (3.96,5.62)	4.44 (3.65,5.24)	3.64 (2.90,4.38)	3.19 (2.48,3.91)	2.25 (1.56,2.94)
25	Females	4.75 (3.63,5.88)	6.25 (5.04,7.45)	5.57 (4.41,6.74)	3.81 (2.71,4.90)	2.80 (1.72,3.88)	0.88 (0.00,1.97)
30	Females	4.50 (3.07,5.92)	6.73 (5.21,8.26)	5.80 (4.34,7.27)	3.00 (1.57,4.42)	1.60 (0.14,3.06)	0.00 (0.00,1.41)
15	Total	0.82 (0.64,1.00)	0.92 (0.71,1.13)	0.87 (0.68,1.06)	0.76 (0.59,0.93)	0.70 (0.53,0.87)	0.58 (0.40,0.76)
20	Total	2.41 (2.01,2.81)	2.79 (2.35,3.22)	2.61 (2.19,3.03)	2.19 (1.80,2.58)	1.95 (1.57,2.32)	1.43 (1.07,1.80)
25	Total	3.70 (3.01,4.40)	4.51 (3.78,5.24)	4.14 (3.43,4.86)	3.20 (2.51,3.89)	2.65 (1.97,3.33)	1.55 (0.87,2.24)
30	Total	4.42 (3.37,5.47)	5.91 (4.75,7.06)	5.34 (4.24,6.44)	3.21 (2.18,4.24)	1.92 (0.88,2.96)	0.00 (0.00,1.12)

Estimates are not very sensitive to plausible time-gradients of age-specific prevalence at ages where the prevalence is relatively low. The impact – as would be expected given the assumption of exponential increase or decline – is substantial at ages with higher estimated prevalence, especially females over 20 years of age. Model-based estimates of age-specific prevalence suggest modest and linear declines at most ages in the range of interest, with a small positive gradient at very young ages (likely reflecting the ageing-in of perinatally-infected individuals, and therefore not likely to reflect higher incidence in this population) and small negative gradient at the ages with the highest incidence, indicating that incidence may be very slightly over-estimated if such a decline is taking place in the surveyed population.
